# Associations between legacy and emerging per and polyfluoroalkyl substances (PFAS) bioaccumulation and clinicopathological characteristics in breast cancer

**DOI:** 10.3389/fpubh.2026.1879297

**Published:** 2026-08-18

**Authors:** Bei Sun, Zhanhua Gao, Xiyue Liu, Gexue Liu, Shoujun Wang, Jinpu Liu, Junyi Wang, Haiyang Zhang, Mingqing Zhang, Chong Chen, Feng He, Dongzhi Hu, Jie Hao

**Affiliations:** 1 Institute of Medical Engineering and Translational Medicine, Tianjin University, Tianjin, China; 2 Department of Pharmacy, The Second Hospital of Tianjin Medical University, Tianjin, China; 3 Tianjin Union Medical Center, The First Affiliated Hospital of Nankai University, Tianjin Medical University Cancer Institute and Hospital, National Clinical Research Center for Cancer, Tianjin, China

**Keywords:** bioaccumulation, breast cancer, clinicopathological characteristics, lipid metabolism, PFAS, tumor biomarkers

## Abstract

**Inroduction:**

Per and polyfluoroalkyl substances (PFAS) are persistent environmental contaminants with endocrine-disrupting and carcinogenic potential. However, their bioaccumulation profiles and clinical relevance in human breast cancer remain largely unexplored.

**Methods:**

In this preliminary study we quantified the absolute concentrations of 33 PFAS congeners (ng/g) in paired tumour and adjacent non-tumorous tissues from 11 breast cancer patients (*n* = 11) using LC–MS/MS to evaluate their tissue distribution and clinical correlations.

**Results:**

The detection rates for six compounds (HFPOTA, PFOA, PFNA, PFUnDA, PFOS, and 6:2Cl-PFESA) were greater than 40%. In paired samples, several congeners showed higher concentrations in tumour than in adjacent non-tumorous tissue; using a two-sided exact Wilcoxon signed-rank test with Hodges–Lehmann effect sizes, tumour enrichment remained significant after Benjamini–Hochberg correction for HFPOTA (*q* = 0.041), PFNA (*q* = 0.047) and PFUnDA (*q* = 0.047). HFPOTA showed by far the highest tissue burden (median 654 ng/g). Clinically, PFOS concentrations were descriptively higher in Luminal B than in Luminal A tumours and were positively correlated with the proliferation marker Ki-67; a high 6:2Cl-PFESA–PR rank correlation was observed in four samples but was not statistically significant on the raw p-value (p ≈ 0.051) and is exploratory. Bioinformatic analyses indicated that computationally predicted shared target genes of PFAS and breast cancer are enriched in the PPAR signaling pathway, lipid metabolism, and steroid hormone biosynthesis.

**Discussion:**

Collectively, PFAS bioaccumulation was associated with breast cancer clinicopathological characteristics. Because the target genes are predicted rather than experimentally validated and the cohort is small (*n* = 11), these are exploratory, hypothesis-generating findings that require validation in larger cohorts. This study underscores the need to reassess the biosafety of novel PFAS alternatives and provides new insights into the environmental etiology of breast cancer.

## Introduction

Per and polyfluoroalkyl substances (PFAS) are a class of synthetic fluorine-containing chemicals that are widely distributed in the environment (1, 2). Due to their strong chemical stability and bioaccumulative properties, PFAS have emerged as critical global environmental pollutants (3). These compounds have been detected in human blood, breast milk, and various tissues, posing potential threats to human health (4–6). These compounds have drawn significant public health concern due to their documented associations with potential carcinogenic activities (7, 8). Accumulating evidence suggests that exposure to PFAS can trigger endocrine disruption, oxidative stress, immunotoxicity, and metabolic dysregulation, potentially contributing to several chronic diseases (9–12).

Importantly, the tissue distribution of PFAS is governed largely by protein binding rather than by simple lipid solubility: PFAS are predominantly proteinophilic and bind with high affinity to serum albumin and to intracellular and liver fatty-acid–binding proteins (FABP/L-FABP), and can engage fatty-acid transporters and peroxisome proliferator-activated receptor (PPAR) signalling. Their pharmacokinetics and tissue partitioning therefore differ markedly among congeners. Nonetheless, unlike blood or milk, human breast tissue has seen few direct PFAS measurements, limiting our understanding of target-organ dose and the congener-specific partitioning into the breast microenvironment.

Breast cancer is the most common malignancy among women worldwide (13). Despite advances in screening and treatment, its etiology remains complex (14). Environmental pollutants, including PFAS, have drawn increasing attention for their possible role in breast cancer development (15, 16). While epidemiological and experimental studies suggest an association between PFAS exposure and breast cancer risk (17, 18), systematic analyses of PFAS in breast tumour tissues and matched non-tumorous adjacent tissues remain scarce, and the relationship between PFAS tissue levels and clinicopathological parameters (stage, grade, lymph-node metastasis, proliferation, molecular subtype) remains unclear.

This study addresses these gaps by measuring multiple PFAS in paired breast cancer tissue samples. By integrating clinicopathological parameters and tumour biomarkers, we explore associations between PFAS bioaccumulation and breast cancer histological features, molecular subtypes, and cellular proliferation activity, and use functional enrichment of PFAS-related target genes to generate mechanistic hypotheses for future study, offering new perspectives for evaluating health risks associated with emerging PFAS substitutes.

## Materials and methods

### Human samples

Patients with breast cancer were recruited from the First Affiliated Hospital of Nankai University. The study included 11 patients with stage I–IV breast cancer who were scheduled for surgical tumour resection; patients with other malignancies or chronic inflammatory diseases were excluded. Informed consent was obtained from all participants, and the study was approved by the hospital’s ethics review board (E20250197). Patient demographic and clinicopathological characteristics are summarised in Table 1. Among environmental exposure determinants, only residential location was recorded (nine patients resided in Tianjin and two in Hebei province); diet, drinking-water source and occupation were not collected and are acknowledged as unmeasured confounders.

**Table 1:** Baseline clinicopathological characteristics of 11 breast cancer patients.

Variable	Category/Statistics	Value (*N* = 11)/(%)
Gender	Female	11 (100.0%)
Age group (years)	≥55	8 (72.7%)
	<55	3 (27.3%)
Menopausal status	Postmenopausal	11 (100.0%)
Residence (province)	Tianjin	9 (81.8%)
	Hebei	2 (18.2%)
Molecular subtype	Luminal A	3 (27.3%)
	Luminal B (HER2−)	4 (36.4%)
	Luminal B (HER2+)	4 (36.4%)
Tumor size (T stage)	T1	2 (18.2%)
	T2	9 (81.8%)
Node status (N stage)	N0	5 (45.5%)
	N1	2 (18.2%)
	N2	3 (27.3%)
	N3	1 (9.1%)
Metastasis (M stage)	M0	10 (90.9%)
	M1	1 (9.1%)
Tumor stage	Stage I	1 (9.1%)
	Stage II	6 (54.5%)
	Stage III	3 (27.3%)
	Stage IV	1 (9.1%)
Histologic grade	Grade 2	8 (72.7%)
	Grade 3	3 (27.3%)
Neoadjuvant therapy	None (treatment-naive)	11 (100.0%)
Receptor status		
ER status	Positive	11 (100.0%)
PR status	Positive	11 (100.0%)
HER2 status	Negative	7 (63.6%)
	Positive	4 (36.4%)
Personal history		
Smoking history	No	10 (90.9%)
	Yes	1 (9.1%)
Drinking history	No	11 (100.0%)
Metastatic status		
Lymph node metastasis	No	5 (45.5%)
	Yes	6 (54.5%)
Distant metastasis	No	10 (90.9%)
	Yes	1 (9.1%)
Weight (kg)	Median (IQR)	58.5 (54.2–63.8)

SD, standard deviation; IQR, interquartile range; ER, oestrogen receptor; PR, progesterone receptor; HER2, human epidermal growth factor receptor 2. Age was dichotomised at 55 years, a widely used breast-cancer risk threshold. Residence is the only environmental exposure determinant recorded; diet, drinking-water source and occupation were not collected.

### Sample preservation

Breast tumour tissues and adjacent non-tumorous tissues (ANT; located ≥ 5 cm from the tumour margin) were obtained from surgical resection specimens. All samples were handled under plastic-free conditions to avoid contamination, immediately placed in sterile glass containers, and stored at −20 °C until analysis.

### Solid-phase extraction and LC–MS/MS quantification

A defined wet tissue mass was extracted three times with 2 mL of methanol and centrifuged. Supernatants were combined, diluted with 200 mL of ultrapure water, and purified by HLB SPE (pre-conditioned with 10 mL methanol and 10 mL ultrapure water; washed with 4 mL of 25 mM ammonium acetate; eluted with 4 mL methanol then 4 mL ammonia/methanol). Eluates were evaporated to near dryness under nitrogen and reconstituted in methanol, filtered, and analysed by LC–MS/MS. Quantitative analysis was performed by a CMA-accredited laboratory on a SCIEX ExionLC coupled to a SCIEX Triple Quad 5500+ mass spectrometer, operated in negative-ion electrospray ionisation with scheduled multiple reaction monitoring (MRM); the MRM transitions and retention times for the six core congeners are listed in Table S2. All 33 congeners were quantified against 12-point external-standard calibration curves (0.1–500 ng/mL) and reported as ng/g (wet weight); isotope-labelled internal standards were not used. Quality control comprised a procedural blank (below detection for all analytes), a 10 ng/mL calibration-verification standard and a 20 ng/mL matrix spike, with recoveries of 85–113% for the six core congeners (Table S2). Instrument detection limits were 2 ng/mL for HFPOTA, 0.2 ng/mL for PFODA and 0.1 ng/mL for the remaining analytes. The complete per-sample concentration matrix for all 33 congeners is provided in Table S1.

### Chemical structure retrieval and toxicological prediction

The 2D/3D structures, canonical SMILES, and descriptors of the six core PFAS (HFPOTA, PFOA, PFNA, PFUnDA, PFOS, 6:2Cl-PFESA) were obtained from PubChem and submitted to ProTox 3 and ADMETlab 3.0 for in silico toxicological profiling across carcinogenicity, mutagenicity, nephrotoxicity, hepatotoxicity, and ecotoxicity. These in silico predictions are derived from structure–activity models; they are not tissue-specific or dose-resolved and are used only to provide preliminary hazard context, not as evidence of carcinogenicity in human breast tissue.

### Bioinformatics

Breast cancer-related genes were obtained from TCGA-BRCA differential expression (adjusted *p* < 0.05, |log₂FC| > 1; 11,532 genes). PFAS-related targets were collected from CTD and SwissTargetPrediction, merged and standardised via UniProt (837 genes). Intersection yielded 392 overlapping genes, subjected to GO, KEGG and Reactome enrichment (clusterProfiler; adjusted *p* < 0.05).

### Statistical analysis

Analyses used R (version 4.5.2). Continuous variables were assessed for normality using the Shapiro–Wilk test; given non-normal paired data, non-parametric methods were used. Values below the limit of detection were substituted with LOD/√2 before analysis; all other values were used as reported (Table S1). The two-sided exact Wilcoxon signed-rank test was used for paired tumour-versus-adjacent-non-tumorous comparisons of PFAS, and Spearman’s correlation for associations between variables. For each paired comparison the effect size was expressed as the Hodges–Lehmann median tumour/adjacent fold-change with an exact 95% confidence interval (Table S3). To control the false-discovery rate across the multiple congeners tested, *p*-values were adjusted with the Benjamini–Hochberg procedure and adjusted *q*-values are reported; two-sided significance was set at *q* < 0.05. Significance is marked on the paired-comparison figure with an asterisk (*) denoting *q* < 0.05. Given the small cohort (*n* = 11) and the low power of some comparisons, all findings are interpreted as exploratory; for each comparison we report the effective sample size, the effect size with 95% CI, the raw *p* value and the adjusted q value. Subgroup comparisons across clinicopathological features involve very small and uneven groups and are therefore presented descriptively and are not used to support inferential conclusions.

## Results

### Patient demographics and clinicopathological characteristics

The baseline characteristics of the 11 female patients are summarised in Table 1. All were hormone receptor-positive (ER and PR positivity 100%). The cohort was predominantly Luminal B (72.7%; HER2-negative 36.4%, HER2-positive 36.4%), with 27.3% Luminal A. Most patients had T2 tumours (81.8%) and Stage II–III disease (81.8%); lymph-node metastasis was present in 54.5%, and one case (9.1%) had distant metastasis. Most tumours were Grade 2 (72.7%), the remainder Grade 3 (27.3%).

### Detection profiles, structure, and toxicological predictions of PFAS in paired tumour and adjacent non-tumorous tissues

**Figure 1:**
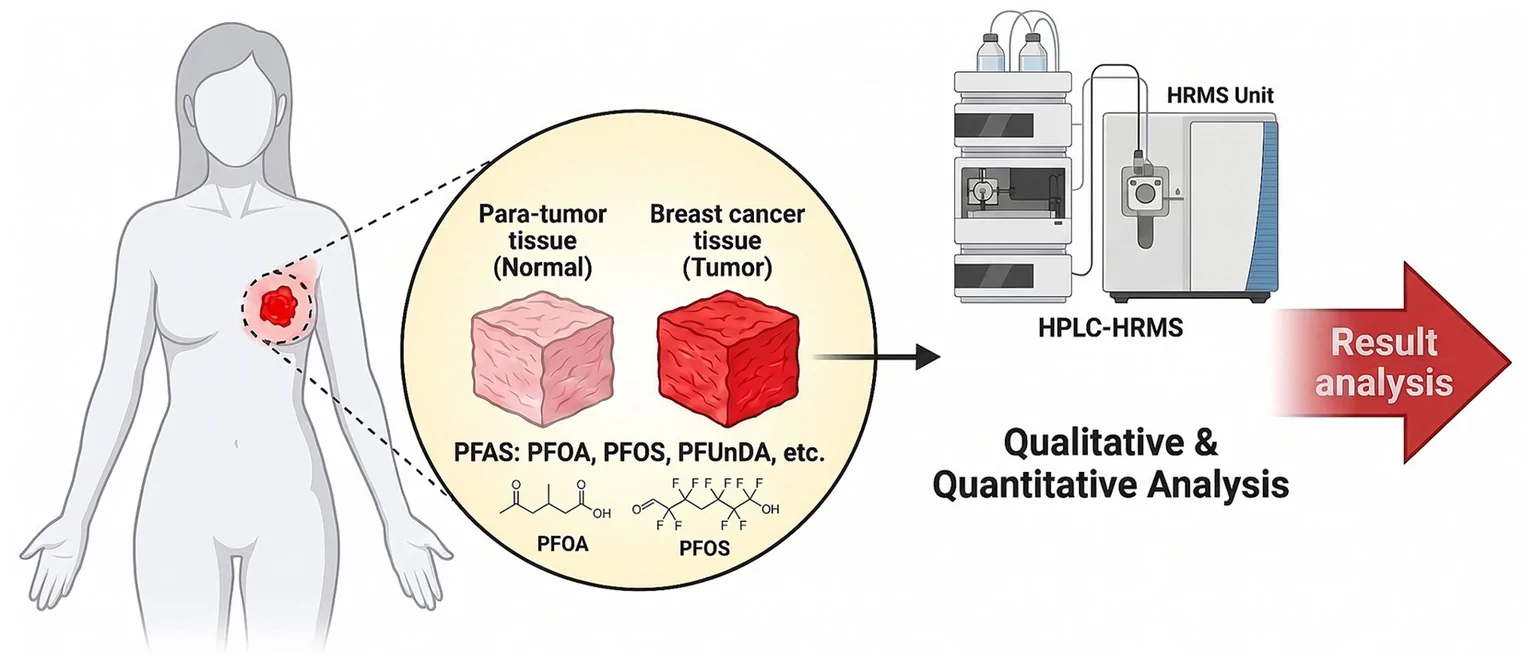
Schematic diagram illustrating the study workflow: paired tumour and adjacent non-tumorous (ANT) tissues were collected from 11 breast cancer patients, followed by PFAS analysis using LC–MS/MS and subsequent result interpretation.

**Figure 2:**
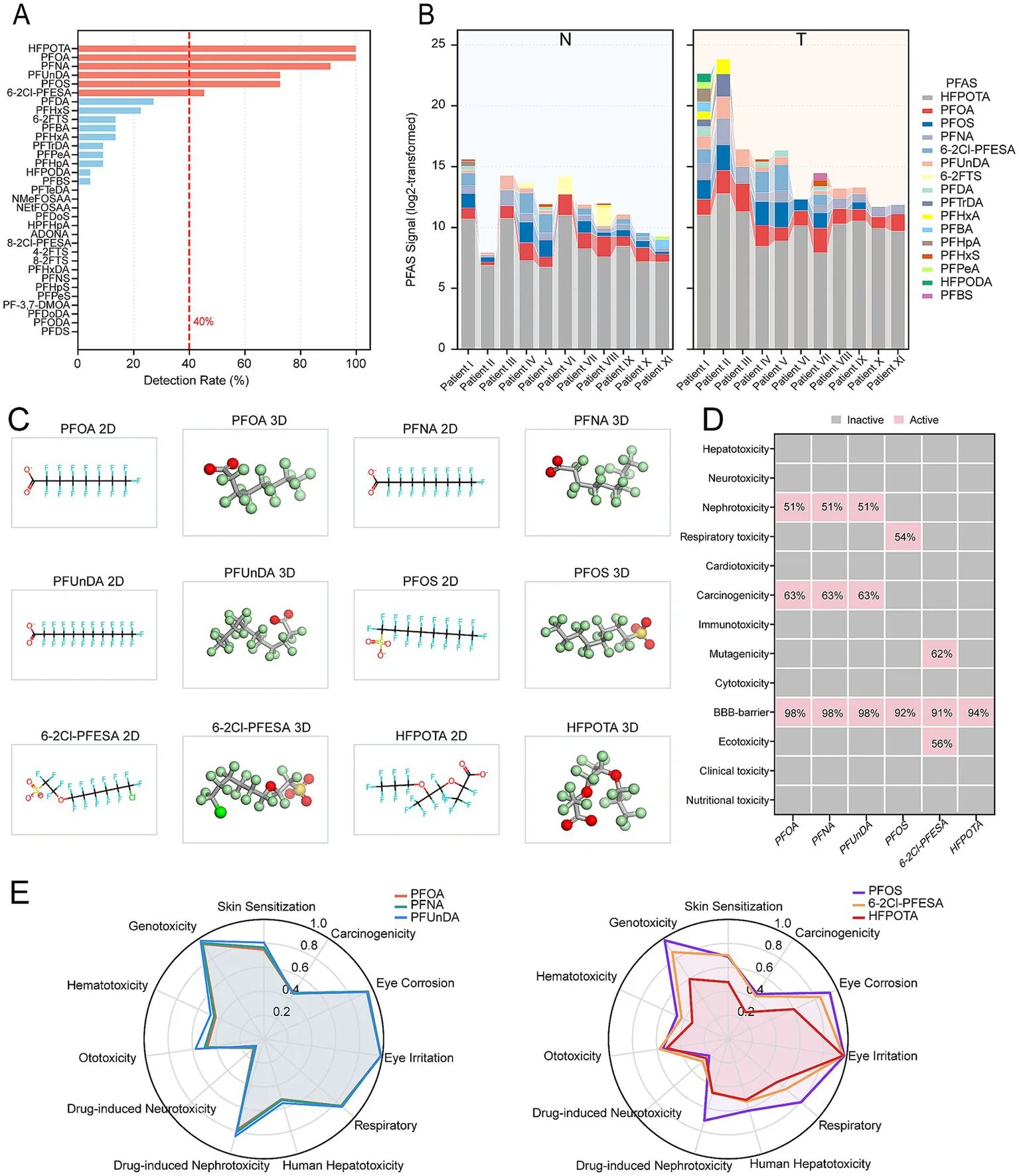
Profiles, structural characteristics, and toxicological assessments of PFAS detected in human breast cancer tissues. **(A)** Detection rates of PFAS compounds in 22 tissue samples. **(B)** Absolute PFAS concentrations (ng/g wet weight) in adjacent non-tumorous and tumour tissues. **(C)** 2D and 3D molecular structures of the six representative PFAS (PFOA, PFNA, PFUnDA, PFOS, 6:2Cl-PFESA, HFPOTA). **(D)** In silico toxicological risk assessment across multiple endpoints. **(E)** Radar charts comparing the multi-dimensional toxicological profiles.

We collected adjacent non-tumorous and tumour tissues from 11 breast cancer patients (22 samples), analysed by LC–MS/MS (Figure 1). Of 33 targeted congeners (Figure 2A), HFPOTA, PFOA, PFNA, PFUnDA, PFOS and 6:2Cl-PFESA were detected in >40% of samples and were prioritised for quantitative comparison; the complete 33-congener concentration profile, including low-detection congeners, is provided in Table S1. Comparison of absolute concentrations (ng/g) in adjacent non-tumorous (N) and tumour (T) tissues showed these PFAS in both tissue types, with preferential accumulation of specific congeners in tumour tissue (Figure 2B).

Figure 2C shows the 2D/3D structures of the six core PFAS. Structure-based in silico screening (Figures 2D,E) flagged predicted hazards such as carcinogenicity/nephrotoxicity for PFOA, PFNA and PFUnDA, and mutagenicity/ecotoxicity for 6:2Cl-PFESA and HFPOTA. These predictions are computational and are presented as preliminary hazard context only.

### Comparison of PFAS levels in paired adjacent non-tumorous and tumour tissues

**Figure 3:**
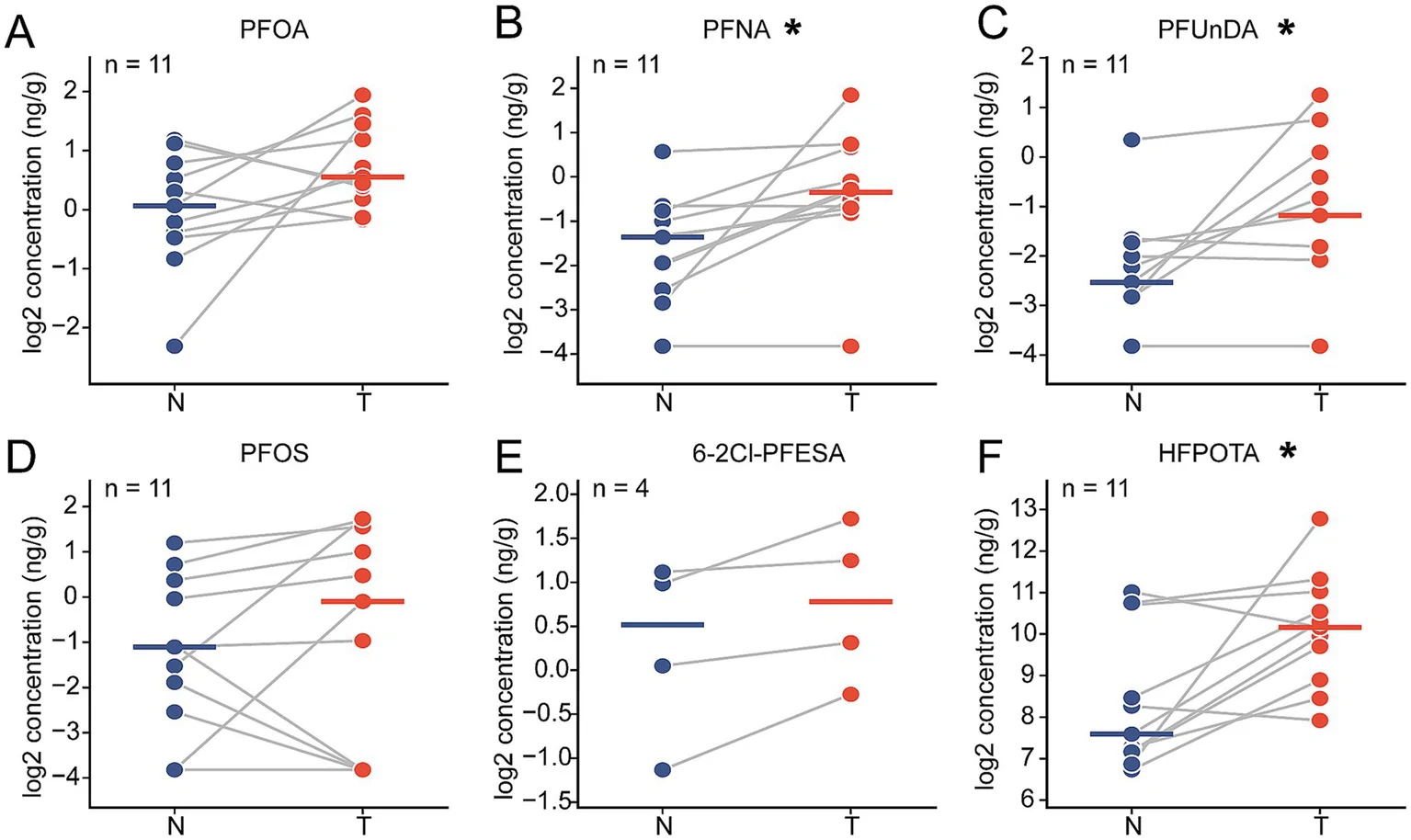
Comparison of PFAS concentrations in paired adjacent non-tumorous (N) and tumour (T) breast tissues (*n* = 11). **(A–F)** Paired line plots of absolute concentrations (ng/g wet weight) for PFOA, PFNA, PFUnDA, PFOS, 6:2Cl-PFESA, and HFPOTA; significance by two-sided exact Wilcoxon signed-rank test with Benjamini–Hochberg correction (asterisk denotes *q* < 0.05).

Using a two-sided exact Wilcoxon signed-rank test on the paired log2 concentrations (*n* = 11), tumour concentrations were significantly higher than in adjacent non-tumorous tissue for HFPOTA (fold-change 3.02, 95% CI 1.49–6.84; *p* = 0.007), PFNA (1.88, 1.15–4.28; *p* = 0.016) and PFUnDA (1.75, 1.03–4.13; *p* = 0.023) (Figures 3B,C,F). After Benjamini–Hochberg correction across the six core congeners, all three remained significant (HFPOTA *q* = 0.041; PFNA *q* = 0.047; PFUnDA *q* = 0.047). PFOA showed a non-significant trend (fold-change 1.36, 0.77–2.93; *p* = 0.413; *q* = 0.496; Figure 3A); no significant difference was detected for PFOS (1.15, 0.48–3.50; *p* = 0.732; *q* = 0.732; Figure 3D) or, given limited detection (*n* = 4 paired), for 6:2Cl-PFESA (fold-change 1.41; *p* = 0.125; *q* = 0.188; Figure 3E). Effect sizes (Hodges–Lehmann tumour/adjacent fold-changes with 95% CI) for all six congeners are provided in Table S3.

Collectively, HFPOTA, PFNA and PFUnDA—the congeners that remained significant after multiple-testing correction—are preferentially accumulated in breast cancer tissues, this within-patient tumour-versus-ANT enrichment is exploratory and does not establish a role in tumorigenesis.

### Association between PFAS levels and breast cancer clinicopathology

**Figure 4:**
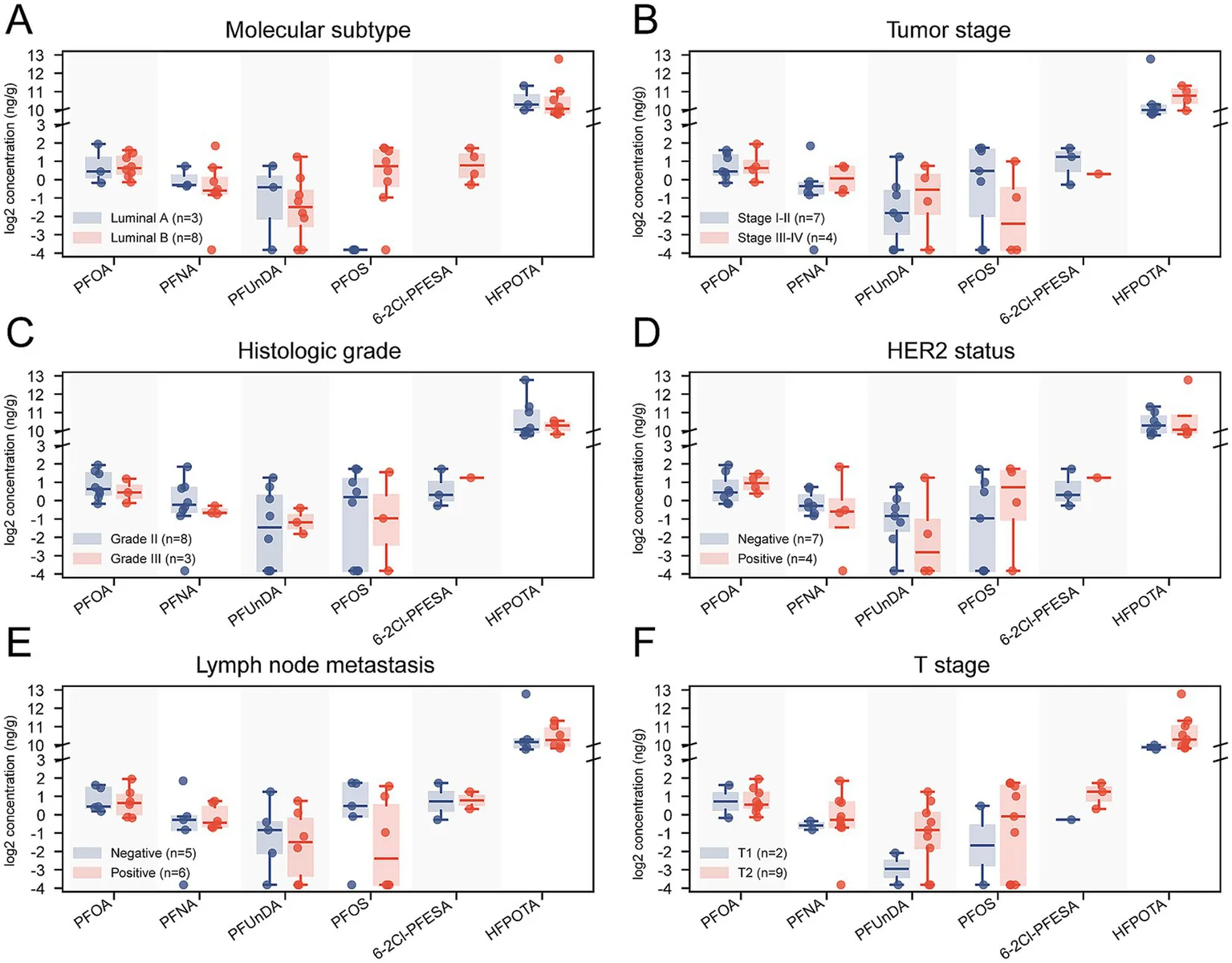
The comparative analysis of 6 PFASs concentrations and clinicopathological characteristics of human breast cancer. **(A)** PFAS levels in Luminal A and Luminal B Breast Cancer Subtypes. **(B)** Tumor clinical stages. **(C)** Histological grading. **(D)** HER2 status. **(E)** Lymph node metastasis. **(F)** T stage.

Across clinicopathological subgroups (Figures 4A–F), PFOS levels were higher in Luminal B than Luminal A tumours (Luminal A, *n* = 3; Luminal B, *n* = 8) (Figure 4A). Because PFAS may exhibit xenoestrogenic effects, this is consistent with an association between PFOS accumulation and the Luminal B phenotype; however, given the very small and uneven subgroups, it should be regarded as a preliminary observation. The tissue burden of HFPOTA, a novel alternative, was exceptionally high (median 654 ng/g; range 106–7,013 ng/g), two to three orders of magnitude above every legacy congener measured (all medians < 1.4 ng/g). This identifies HFPOTA as the most abundant PFAS measured in these tissues and warrants further toxicological and epidemiological investigation.

### Correlations between tumour PFAS levels and clinical markers

**Figure 5:**
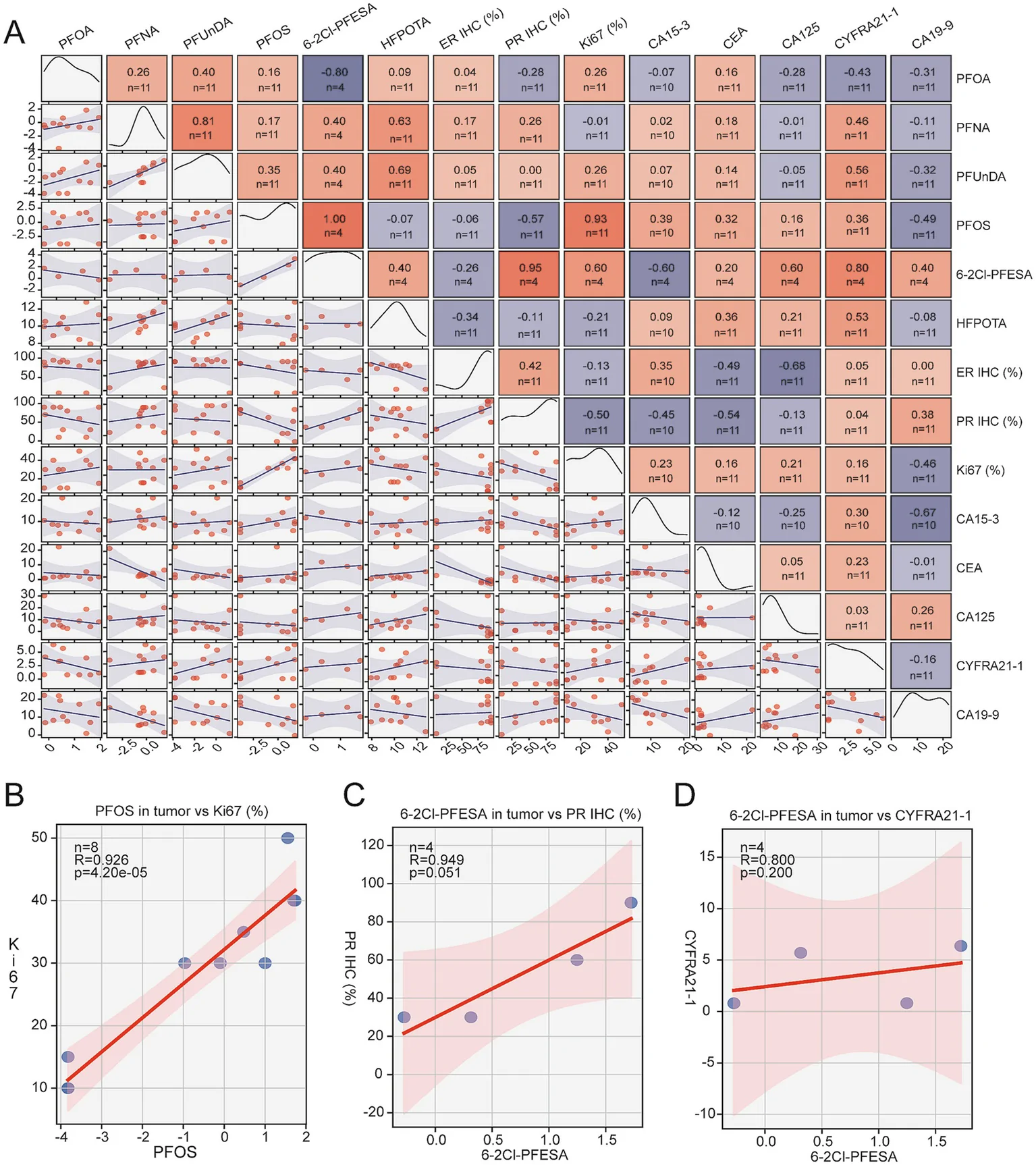
Correlations between intratumoral PFAS concentrations and clinical biomarkers. **(A)** Correlation matrix of the six core PFAS and tumour biomarkers (per-cell Spearman *r* and *p*). **(B–D)** Individual scatter plots with linear regression lines detailing specific associations: **(B)** PFOS vs. Ki-67 (*n* = 8; *r* = 0.93). **(C)** 6:2Cl-PFESA vs. PR IHC (*n* = 4; *r* = 0.95). **(D)** 6:2Cl-PFESA vs. CYFRA21-1 (*n* = 4; *r* = 0.80). In all scatter plots the *x*-axis is the log2 PFAS concentration and the *y*-axis is the biomarker. Shaded regions represent 95% confidence intervals. CEA, carcinoembryonic antigen; CA19-9, carbohydrate antigen 199; PR, progesterone receptor; and data are analyzed by Spearman’s correlation analysis.

The legacy congener PFOS showed a strong positive correlation with the Ki-67 proliferation index (*r* = 0.93; *n* = 8; Figure 5B). This is consistent with an association between PFOS accumulation and tumour proliferative activity; however, given the cross-sectional design and small sample, causality cannot be inferred and reverse causation (preferential uptake by highly proliferative tumours) is an equally plausible explanation. The novel alternative 6:2Cl-PFESA showed high correlations with PR (*r* = 0.95) and CYFRA21-1 (*r* = 0.80), but these were based on only four tumour samples each tumour samples, respectively, did not survive correction, and are reported strictly as preliminary, hypothesis-generating observations requiring validation.

### Genomic intersection and functional enrichment analysis

**Figure 6:**
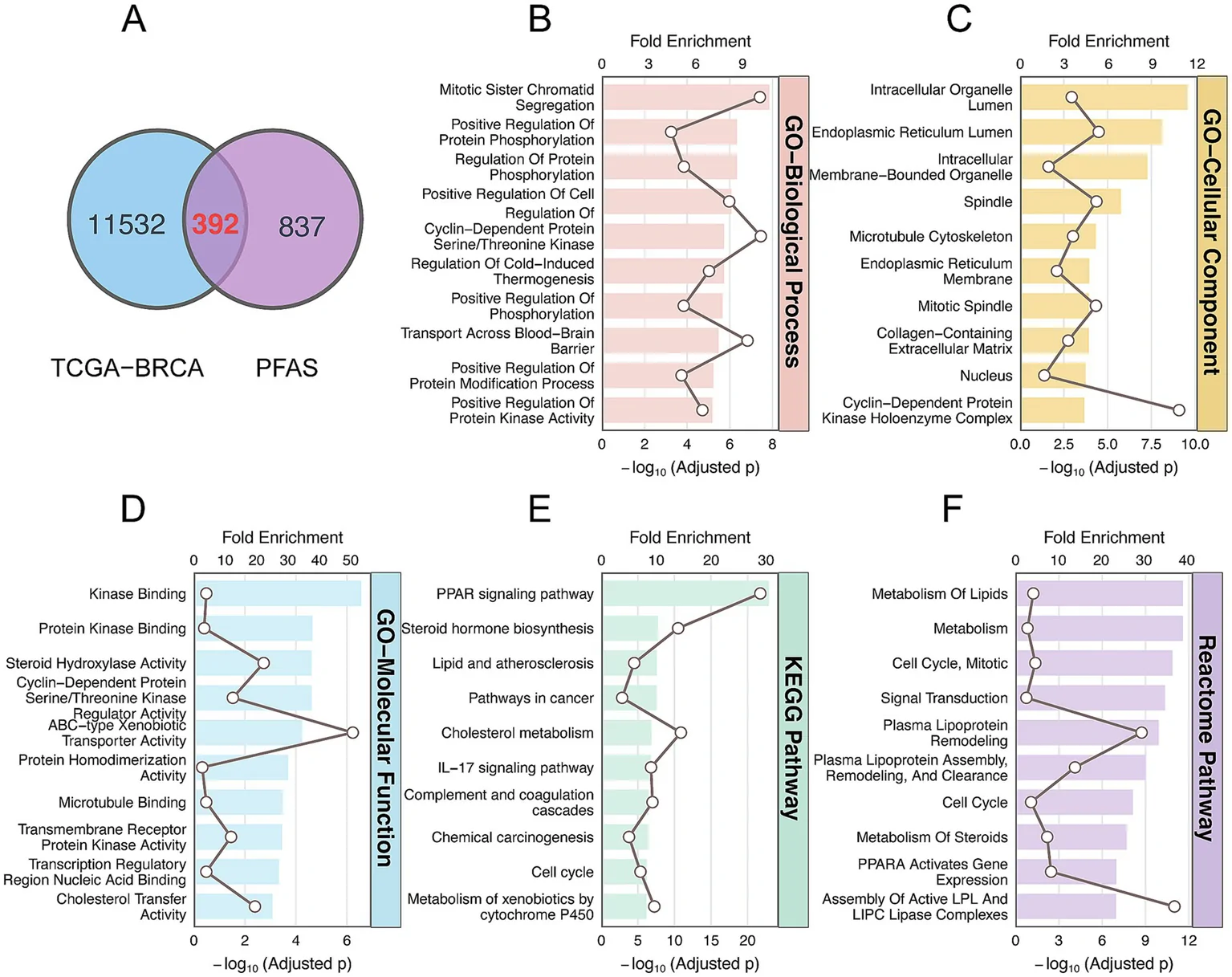
Intersection and functional enrichment analysis of PFAS-associated genes and breast cancer (TCGA-BRCA) related genes. **(A)** Genes of TCGA-BRCA cointeracting with perfluorinated compounds. **(B–D)** Gene Ontology functional enrichment analysis of the 392 intersecting genes: **(B)** Biological process (BP) enrichment analysis; **(C)** Cellular component (CC) enrichment analysis; **(D)** Molecular function (MF) enrichment analysis; **(E)** Kyoto Encyclopedia of Genes and Genomes (KEGG) pathway enrichment analysis; **(F)** Reactome pathway enrichment analysis.

Intersection analysis identified 392 overlapping genes between TCGA-BRCA differentially expressed genes and computationally predicted PFAS-related targets (Figure 6A). GO analysis (Figures 6B–D) highlighted mitotic sister chromatid segregation, protein phosphorylation, kinase binding, steroid hydroxylase activity and ABC-type xenobiotic transporter activity; KEGG identified the PPAR signaling pathway as most enriched (Figure 6E); Reactome highlighted lipid metabolism, cell-cycle regulation, steroid metabolism and PPARA-mediated gene expression (Figure 6F). Because the PFAS targets are computationally predicted and were not validated in our own cohort, this analysis is strictly hypothesis-generating: it nominates pathways for future functional testing and does not demonstrate that the detected PFAS regulate these genes in these patients.

## Discussion

Our study investigates the statistical associations of exposure to legacy and novel PFAS with clinicopathological characteristics and tumour markers in breast cancer, integrating tissue-targeted metabolomics, in silico toxicity prediction, and toxicogenomic bioinformatics. Overall, our cross-sectional findings show that independent in silico overlap and enrichment analyses nominated lipid-metabolism and PPAR-related pathways; these analyses were not statistically linked to the measured tissue PFAS concentrations and were not validated in this cohort; these observations are hypothesis-generating and do not establish that PFAS cause, initiate or promote breast cancer.

Ki-67 reflects proliferation, and high levels associate with aggressive behaviour and poorer prognosis (19, 20); Luminal B tumours have higher proliferation than Luminal A (21, 22). In our cohort, PFOS accumulation correlated with Ki-67 and was higher in Luminal B. This suggests PFAS may be retained in the tumour environment (23); however, the direction of this association cannot be established from cross-sectional data.

As a novel PFECA, HFPOTA is widely detected in the environment (24). Prior work reported strong proliferation-promoting effects at low concentrations, hepatocyte toxicity exceeding PFOA, and high liver accumulation/hepatotoxicity in mice (25–27). This study reports a high detection rate and concentrations of the novel HFPOTA (detected in 100% of samples), together with detectable 6:2Cl-PFESA (45% of samples) in breast tissue, providing direct measurements in breast tissue rather than serum-based exposure estimates; these alternatives require additional toxicological study before their safety as substitutes can be established (28–30).

Mechanistically, PFAS consist of a hydrophobic fluorocarbon backbone and a hydrophilic polar head group (31). Contrary to a purely lipophilic view, PFAS are predominantly proteinophilic: they bind with high affinity to serum albumin and to intracellular and liver fatty-acid–binding proteins (FABP/L-FABP), so their tissue partitioning is governed largely by protein binding rather than by simple lipid dissolution (32, 33). PFAS can also activate PPARα (34, 35); because breast cancer arises from a lipid-rich microenvironment dependent on lipid metabolic reprogramming (36, 37), accumulation likely reflects the combined protein and lipid-rich tumour microenvironment. Our bioinformatic overlap suggests, as a hypothesis for future functional testing, that PFAS may act partly through PPAR and lipid-metabolism–related pathways; because these targets are predicted and unvalidated in our cohort, this does not establish a mechanism (38, 39).

This study has several limitations. First, the sample size is small (*n* = 11), limiting power and widening confidence intervals, so the study is exploratory and hypothesis-generating and requires validation in larger, independent cohorts. Second, reverse causation is a leading alternative explanation: proliferating, metabolically active tumours up-regulate fatty-acid transporters and binding proteins and may preferentially sequester circulating PFAS, so higher tumour PFAS levels and their marker correlations may reflect tumour biology driving PFAS uptake rather than the reverse. Third, “adjacent non-tumorous” tissue may already harbour field-cancerization–related changes and does not represent a truly unexposed baseline. Fourth, several exposure and lifestyle determinants were not captured: diet, drinking-water source and occupation were not recorded, and body-mass index could not be calculated because height was not available; these remain unmeasured confounders. Finally, our targeted panel likely underestimates the broader exposome. Future studies should focus on HFPOTA and 6:2Cl-PFESA to test the molecular mechanisms by which they may modulate the PPAR and steroid-hormone axes in breast cancer.

## Conclusion

This study characterized the bioaccumulation of legacy and emerging PFAS in paired breast cancer and adjacent non-tumorous tissues, reporting absolute concentrations (ng/g). Six core PFAS were frequently detected, with HFPOTA, PFNA and PFUnDA showing significant tumour-preferential accumulation after multiple-testing correction; HFPOTA exhibited an exceptionally high tissue burden. PFAS levels were associated with clinicopathological features, as PFOS showed a descriptive subtype pattern (higher in Luminal B) and a correlation with Ki-67, whereas the high 6:2Cl-PFESA–PR correlation estimate, based on six samples, was not statistically significant (raw *p* ≈ 0.051) and is exploratory. Bioinformatic analysis nominated the PPAR signaling pathway, lipid metabolism, and steroid hormone biosynthesis as candidate pathways. Our findings provide preliminary evidence of associations between PFAS bioaccumulation and clinicopathological characteristics, highlighting the need to reassess the biosafety of novel PFAS alternatives and supporting further research on the environmental etiology of breast cancer.

## Data Availability

The original contributions presented in the study are included in the article/Supplementary material, further inquiries can be directed to the corresponding authors.
