# Supplementary material for: Associations between legacy and emerging per and polyfluoroalkyl substances (PFAS) bioaccumulation and clinicopathological characteristics in breast cancer
Source: Front Public Health. 2026 Aug 18;14:1879297. doi: 10.3389/fpubh.2026.1879297 (PMC13529988; doi:10.3389/fpubh.2026.1879297)
Supplement: Supplementary file 1 [file Supplementary_file_1.docx]

**Supplementary Analysis — Independent Population-Level Association Between Serum PFAS and Breast-Cancer History (NHANES 2003–2018)**

*Supplement to Manuscript ID 1879297, Frontiers in Public Health (Environmental Health and Exposome)*

**Purpose and scope.** This supplementary analysis provides independent, population-level context for the paired-tissue study reported in the main manuscript. It is kept deliberately separate from the tissue data and is not merged with them. Serum concentration and tissue accumulation are distinct quantities that answer different questions: a serum measurement reflects recent systemic exposure, whereas the tissue measurement in the main study reflects the PFAS burden sequestered within resected tumour and adjacent non-tumorous tissue. A population-level serum–disease association therefore neither confirms nor refutes the within-patient tumour enrichment reported in the main manuscript. We present this analysis to show that the two layers are not interchangeable, and to delimit the specific contribution of the tissue study.

**S1. Supplementary Methods**

**Data source.** We merged eight continuous NHANES cycles (2003–2004 through 2017–2018). Public-use serum PFAS, demographic, reproductive-health, body-measures, biochemistry and medical-conditions files were linked on the participant identifier (SEQN). Source: https://wwwn.cdc.gov/nchs/nhanes.

**Study population.** Eligible participants were women aged ≥20 years; known pregnancies were excluded. Breast-cancer cases were defined by a self-reported physician diagnosis of breast cancer (MCQ230A/B/C = 14); the primary control group comprised women with no reported cancer of any type (MCQ220 = 2). The primary analytic cohort contained 5,963 women with a valid value for at least one primary PFAS (153 breast-cancer cases); the fully adjusted model (Model 2) was based on 4,194 women with complete covariates (105 cases). Figure S1 shows the cohort-selection flow.

**Exposure and survey design.** Four frequently detected congeners were treated as primary exposures (PFOA, PFOS, PFNA, PFUnDA) and two as secondary (PFHxS, PFDeA). For 2013–2014, total PFOA and PFOS were reconstructed by summing linear and branched isomers, using the corresponding subsample weight (WTSSBH2Y). All analyses used the NHANES complex survey design: cycle-nested strata (SDMVSTRA) and primary sampling units (SDMVPSU), with 16-year pooled weights formed by dividing each cycle's 2-year PFAS-subsample weight by eight. CDC laboratory values already imputed below the limit of detection were used as released. Because PFUnDA was detected in about 46% of samples, it was modelled as detectable versus non-detectable; the remaining congeners were modelled per doubling of concentration (log2).

**Outcome models.** Survey-weighted logistic regression estimated the association between each PFAS and breast-cancer history. Model 1 adjusted for age (natural spline, df = 3), race/ethnicity and survey cycle. Model 2 added education, income-to-poverty ratio, body-mass index, smoking, alcohol use, parity and menopausal status. The four primary congeners formed one testing family with Benjamini–Hochberg (FDR) correction. Quartile models were fitted only where every quartile contained at least 10 cases. A restricted-cubic-spline dose–response (Figure S3) was estimated for the continuously modelled congeners.

**Sensitivity analyses.** Six pre-specified variations tested robustness: expanded controls (including other cancers), restriction to age ≥40 years, exclusion of participants with any non-breast cancer, additional adjustment for eGFR, exclusion of exposures above the 99th percentile, and 20-fold random hot-deck imputation of missing covariates.

**Software and reproducibility.** Analyses used R with the survey package; lonely PSUs were centred at the grand mean and the random seed was fixed. The full analysis script, intermediate tables and figures are archived alongside the manuscript.

**S2. Supplementary Results**

**Cohort characteristics.** Women with a breast-cancer history were, as expected, considerably older than cancer-free women (mean 66.5 vs 46.7 years) and predominantly postmenopausal (93.0% vs 45.8%), with a higher proportion of former smokers. These strong age and reproductive differences are the main confounders addressed by adjustment (Table SN1).

**Table SN1.** Weighted baseline characteristics of the analytic cohort, by breast-cancer history (NHANES 2003–2018). Percentages and means are survey-weighted; unweighted n is shown for each cell.

| **Characteristic** | **Level** | **Cancer-free** | **Breast cancer** |
| --- | --- | --- | --- |
| Age (years) | Mean (SE) | 46.66 (0.32) (n=5810) | 66.50 (1.31) (n=153) |
| Family income-to-poverty ratio | Mean (SE) | 2.92 (0.04) (n=5286) | 3.25 (0.19) (n=137) |
| Body mass index (kg/m2) | Mean (SE) | 29.00 (0.14) (n=5740) | 29.17 (0.85) (n=152) |
| Live-birth deliveries | Mean (SE) | 1.96 (0.03) (n=5038) | 2.47 (0.17) (n=142) |
| Race/ethnicity | Mexican American | 8.3% (SE 0.7) (n=5810) | 3.2% (SE 1.0) (n=153) |
|  | Other Hispanic | 5.8% (SE 0.5) (n=5810) | 2.3% (SE 0.9) (n=153) |
|  | Non-Hispanic White | 65.6% (SE 1.4) (n=5810) | 82.0% (SE 2.8) (n=153) |
|  | Non-Hispanic Black | 12.3% (SE 0.8) (n=5810) | 6.3% (SE 1.5) (n=153) |
|  | Other/Multi-racial | 7.9% (SE 0.5) (n=5810) | 6.3% (SE 1.7) (n=153) |
| Education | <High school | 15.7% (SE 0.7) (n=5800) | 11.9% (SE 2.9) (n=153) |
|  | High school/GED | 22.6% (SE 0.8) (n=5800) | 25.7% (SE 4.0) (n=153) |
|  | >High school | 61.8% (SE 1.0) (n=5800) | 62.4% (SE 4.3) (n=153) |
| Smoking status | Never | 63.0% (SE 0.9) (n=5806) | 61.0% (SE 5.5) (n=153) |
|  | Former | 18.1% (SE 0.7) (n=5806) | 33.2% (SE 5.4) (n=153) |
|  | Current | 18.9% (SE 0.9) (n=5806) | 5.8% (SE 2.0) (n=153) |
| Alcohol use | No/low | 32.4% (SE 1.0) (n=4850) | 36.4% (SE 5.6) (n=128) |
|  | Current | 67.6% (SE 1.0) (n=4850) | 63.6% (SE 5.6) (n=128) |
| Menstrual status | Menstruating | 54.2% (SE 1.0) (n=5083) | 7.0% (SE 3.0) (n=136) |
|  | Menopause/hysterectomy | 45.8% (SE 1.0) (n=5083) | 93.0% (SE 3.0) (n=136) |

**Serum PFAS by group (unadjusted).** In crude comparisons, geometric-mean serum concentrations were modestly higher in women with a breast-cancer history for several congeners (PFOA 2.73 vs 2.15 ng/mL, p = 0.011; PFHxS 1.54 vs 1.09 ng/mL, p < 0.001). Because cases were on average 20 years older and mostly postmenopausal, these unadjusted differences are strongly confounded by age and do not survive adjustment (Table SN2, and Table SN3 below).

**Table SN2.** Weighted serum PFAS descriptive statistics by breast-cancer history (concentrations in ng/mL). p is the unadjusted survey-weighted group comparison.

| **PFAS** | **Role** | **Group** | **Detect %** | **GM (95% CI)** | **Median (IQR)** | **p** |
| --- | --- | --- | --- | --- | --- | --- |
| PFOA | Primary | Cancer-free | 99.6 | 2.15 (2.06–2.25) | 2.27 (1.27–3.70) | 0.011 |
| PFOA | Primary | Breast cancer | 99.8 | 2.73 (2.25–3.30) | 2.37 (1.73–4.80) | 0.011 |
| PFOS | Primary | Cancer-free | 99.6 | 6.75 (6.42–7.11) | 6.90 (3.50–13.40) | 0.013 |
| PFOS | Primary | Breast cancer | 99.6 | 8.79 (7.12–10.84) | 8.50 (4.80–17.00) | 0.013 |
| PFNA | Primary | Cancer-free | 98.0 | 0.76 (0.72–0.80) | 0.80 (0.50–1.30) | 0.203 |
| PFNA | Primary | Breast cancer | 97.8 | 0.85 (0.70–1.03) | 0.90 (0.50–1.39) | 0.203 |
| PFUnDA | Primary | Cancer-free | 46.0 | 0.15 (0.15–0.16) | 0.14 (0.07–0.20) | 0.161 |
| PFUnDA | Primary | Breast cancer | 57.2 | 0.17 (0.14–0.20) | 0.20 (0.10–0.30) | 0.161 |
| PFHxS | Secondary | Cancer-free | 98.7 | 1.09 (1.04–1.14) | 1.10 (0.60–2.00) | <0.001 |
| PFHxS | Secondary | Breast cancer | 99.9 | 1.54 (1.32–1.80) | 1.40 (1.00–2.40) | <0.001 |
| PFDeA | Secondary | Cancer-free | 73.7 | 0.23 (0.22–0.24) | 0.20 (0.14–0.40) | 0.492 |
| PFDeA | Secondary | Breast cancer | 79.4 | 0.24 (0.20–0.28) | 0.20 (0.14–0.40) | 0.492 |

**Adjusted associations.** After full adjustment, none of the four primary PFAS was associated with breast-cancer history: PFOA OR 1.05 (95% CI 0.75–1.48) per doubling, PFOS 0.92 (0.77–1.11), PFNA 0.97 (0.76–1.23), and PFUnDA 1.49 (0.84–2.66) for detectable versus non-detectable (all p ≥ 0.17; all FDR q ≥ 0.69). Model 1 gave the same picture (Table SN3, Figure S2). Quartile and multi-pollutant models were likewise null, with no monotonic dose–response trend (Figure S3).

**Table SN3.** Survey-weighted association between serum PFAS and breast-cancer history. OR is per doubling of concentration, except PFUnDA (detectable vs non-detectable). FDR q-values apply within the four-congener primary family.

| **PFAS (role)** | **Parameter** | **Model** | **OR (95% CI)** | **p** | **FDR q** |
| --- | --- | --- | --- | --- | --- |
| PFOA (Primary) | Per doubling | Model 1 | 1.11 (0.84–1.45) | 0.455 | 0.889 |
| PFOA (Primary) | Per doubling | Model 2 | 1.05 (0.75–1.48) | 0.773 | 0.778 |
| PFOS (Primary) | Per doubling | Model 1 | 0.99 (0.83–1.18) | 0.910 | 0.910 |
| PFOS (Primary) | Per doubling | Model 2 | 0.92 (0.77–1.11) | 0.395 | 0.778 |
| PFNA (Primary) | Per doubling | Model 1 | 1.05 (0.85–1.30) | 0.667 | 0.889 |
| PFNA (Primary) | Per doubling | Model 2 | 0.97 (0.76–1.23) | 0.778 | 0.778 |
| PFUnDA (Primary) | Detectable vs non-detectable | Model 1 | 1.36 (0.82–2.24) | 0.227 | 0.889 |
| PFUnDA (Primary) | Detectable vs non-detectable | Model 2 | 1.49 (0.84–2.66) | 0.173 | 0.693 |
| PFHxS (Secondary) | Per doubling | Model 1 | 0.98 (0.85–1.14) | 0.823 | — |
| PFHxS (Secondary) | Per doubling | Model 2 | 0.97 (0.83–1.15) | 0.750 | — |
| PFDeA (Secondary) | Per doubling | Model 1 | 1.00 (0.80–1.26) | 0.967 | — |
| PFDeA (Secondary) | Per doubling | Model 2 | 0.98 (0.72–1.33) | 0.879 | — |

**Robustness.** The null result was stable across all six sensitivity analyses and the multiply-imputed analysis; no scenario produced a significant primary association (Table SN4).

**Table SN4.** Model 2 associations across sensitivity scenarios. Cells show OR (95% CI); PFUnDA is detectable vs non-detectable, others per doubling.

| **Scenario** | **PFOA** | **PFOS** | **PFNA** | **PFUnDA (det.)** |
| --- | --- | --- | --- | --- |
| Main Model 2 | 1.05 (0.75–1.48) | 0.92 (0.77–1.11) | 0.97 (0.76–1.23) | 1.49 (0.84–2.66) |
| Expanded controls | 1.02 (0.72–1.43) | 0.92 (0.77–1.10) | 0.95 (0.76–1.20) | 1.53 (0.90–2.61) |
| Age >=40 years | 0.96 (0.71–1.30) | 0.93 (0.77–1.12) | 0.92 (0.74–1.14) | 1.39 (0.78–2.47) |
| Pure breast cancer cases | 1.11 (0.74–1.66) | 0.93 (0.76–1.14) | 0.99 (0.75–1.30) | 1.64 (0.83–3.22) |
| Additional eGFR adjustment | 1.07 (0.76–1.49) | 0.94 (0.78–1.13) | 0.98 (0.77–1.24) | 1.48 (0.83–2.65) |
| Exclude exposure >99th percentile | 0.99 (0.71–1.39) | 0.94 (0.77–1.15) | 0.98 (0.76–1.26) | 1.51 (0.85–2.68) |
| Hot-deck imputation (20×) | 1.08 (0.82–1.42) | 0.97 (0.82–1.15) | 1.02 (0.82–1.27) | 1.30 (0.79–2.13) |

**Supplementary Figures**

**Figure S1.** Cohort-selection flow, from the pooled PFAS subsample to the primary analytic cohort and the Model 2 complete-case cohort.

**Figure S2.** Forest plot of fully adjusted (Model 2) associations between serum PFAS and breast-cancer history. All confidence intervals cross the null.

**Figure S3.** Restricted-cubic-spline dose–response for continuously modelled PFAS; no monotonic trend is evident.

**S3. Interpretation and Limitations**

**Serum versus tissue.** At the population level, serum PFAS was not associated with prevalent breast cancer after adjustment. This does not diminish the paired-tissue observation of congener-specific tumour enrichment reported in the main manuscript. Circulating PFAS concentration need not track the burden sequestered in a particular tissue compartment, so a null serum–disease association and a positive tumour-versus-adjacent tissue contrast are fully compatible. Among the four primary congeners, PFUnDA was the only one whose adjusted serum point estimate pointed in the same direction as its tumour enrichment in the main study (serum OR 1.49 per doubling for detectable versus non-detectable; tumour/adjacent fold-change 1.75), although this serum association was not statistically significant (q = 0.69); the remaining congeners showed no concordant serum trend. The two analyses measure different things; the distinct contribution of the main study is the direct measurement of internal tissue dose, which serum-based epidemiology does not capture.

**Temporality and other limitations.** This is a cross-sectional analysis with a self-reported outcome. For prevalent cases, serum PFAS was measured after diagnosis (45.6% of cases were diagnosed more than 10 years earlier), so the design cannot establish exposure–disease temporality and is open to reverse causation and survivor bias. The analysis therefore serves as independent context, not as a formal validation or refutation of the tissue findings.
