# Supplementary material for: Associations between legacy and emerging per and polyfluoroalkyl substances (PFAS) bioaccumulation and clinicopathological characteristics in breast cancer
Source: Front Public Health. 2026 Aug 18;14:1879297. doi: 10.3389/fpubh.2026.1879297 (PMC13529988; doi:10.3389/fpubh.2026.1879297)
Supplement: Supplementary file 2 [file Supplementary_file_2.docx]

**Supplementary Table S1. Absolute tissue concentrations (ng/g wet weight) of 33 PFAS congeners in paired tumour (T) and adjacent non-tumorous (N) breast tissues from 11 breast cancer patients.**

Values are ng/g wet weight. N.D. = not detected. Instrument detection limits: HFPOTA 2 ng/mL, PFODA 0.2 ng/mL, all other analytes 0.1 ng/mL. Analysis: SCIEX ExionLC coupled to a SCIEX Triple Quad 5500+ (negative-ion ESI, scheduled MRM); CMA-accredited laboratory. Detection rate and median (of detected values) are computed over all 22 samples.

| **Congener** | **696530-N** | **696530-T** | **709395-N** | **709395-T** | **727624-N** | **727624-T** | **511158-N** | **511158-T** | **728794-N** | **728794-T** | **748123-N** | **748123-T** | **533165-N** | **533165-T** | **202721-N** | **202721-T** | **751544-N** | **751544-T** | **718455-N** | **718455-T** | **159919-N** | **159919-T** | **Det. rate** | **Median*** |
| --- | --- | --- | --- | --- | --- | --- | --- | --- | --- | --- | --- | --- | --- | --- | --- | --- | --- | --- | --- | --- | --- | --- | --- | --- |
| PFBA | N.D. | N.D. | N.D. | N.D. | N.D. | N.D. | N.D. | N.D. | 0.669 | N.D. | N.D. | N.D. | N.D. | N.D. | N.D. | 0.737 | N.D. | N.D. | 0.028 | N.D. | N.D. | N.D. | 3/22 (14%) | 0.669 |
| PFPeA | N.D. | N.D. | N.D. | N.D. | N.D. | N.D. | N.D. | N.D. | 0.203 | N.D. | N.D. | N.D. | N.D. | N.D. | N.D. | 0.437 | N.D. | N.D. | N.D. | N.D. | N.D. | N.D. | 2/22 (9%) | 0.320 |
| PFHxA | N.D. | N.D. | N.D. | N.D. | N.D. | N.D. | N.D. | N.D. | N.D. | N.D. | N.D. | N.D. | N.D. | 1.339 | N.D. | 0.549 | N.D. | N.D. | 0.111 | N.D. | N.D. | N.D. | 3/22 (14%) | 0.549 |
| PFHpA | N.D. | N.D. | N.D. | N.D. | N.D. | N.D. | N.D. | N.D. | N.D. | N.D. | N.D. | N.D. | N.D. | N.D. | 0.334 | 1.153 | N.D. | N.D. | N.D. | N.D. | N.D. | N.D. | 2/22 (9%) | 0.744 |
| **PFOA** | **0.767** | **1.134** | **3.052** | **1.437** | **1.730** | **2.275** | **1.240** | **0.887** | **0.561** | **1.646** | **2.283** | **1.311** | **0.201** | **2.742** | **0.864** | **1.466** | **1.047** | **3.841** | **2.176** | **1.361** | **0.718** | **0.912** | **22/22 (100%)** | **1.336** |
| **PFNA** | **0.499** | **0.935** | **0.565** | **0.400** | **0.637** | **0.630** | **0.252** | **0.783** | **0.171** | **0.698** | **N.D.** | **N.D.** | **0.139** | **3.589** | **0.587** | **1.582** | **1.484** | **1.667** | **0.260** | **0.822** | **0.389** | **0.614** | **20/22 (91%)** | **0.622** |
| PFDA | 0.203 | 0.465 | 0.291 | N.D. | N.D. | 0.348 | N.D. | N.D. | N.D. | N.D. | N.D. | N.D. | N.D. | N.D. | 0.242 | 0.759 | N.D. | N.D. | N.D. | N.D. | N.D. | N.D. | 6/22 (27%) | 0.320 |
| **PFUnDA** | **0.215** | **0.559** | **0.236** | **0.249** | **0.318** | **0.285** | **N.D.** | **N.D.** | **N.D.** | **N.D.** | **N.D.** | **N.D.** | **0.139** | **2.376** | **0.173** | **1.067** | **1.271** | **1.683** | **0.141** | **0.753** | **0.301** | **0.441** | **16/22 (73%)** | **0.309** |
| PFDoDA | N.D. | N.D. | N.D. | N.D. | N.D. | N.D. | N.D. | N.D. | N.D. | N.D. | N.D. | N.D. | N.D. | N.D. | N.D. | N.D. | N.D. | N.D. | N.D. | N.D. | N.D. | N.D. | 0/22 (0%) | — |
| PFTrDA | N.D. | N.D. | N.D. | N.D. | N.D. | N.D. | N.D. | N.D. | N.D. | N.D. | N.D. | N.D. | N.D. | 2.654 | N.D. | 0.493 | N.D. | N.D. | N.D. | N.D. | N.D. | N.D. | 2/22 (9%) | 1.573 |
| PFTeDA | N.D. | N.D. | N.D. | N.D. | N.D. | N.D. | N.D. | N.D. | N.D. | N.D. | N.D. | N.D. | N.D. | N.D. | N.D. | N.D. | N.D. | N.D. | N.D. | N.D. | N.D. | N.D. | 0/22 (0%) | — |
| PFHxDA | N.D. | N.D. | N.D. | N.D. | N.D. | N.D. | N.D. | N.D. | N.D. | N.D. | N.D. | N.D. | N.D. | N.D. | N.D. | N.D. | N.D. | N.D. | N.D. | N.D. | N.D. | N.D. | 0/22 (0%) | — |
| PFODA | N.D. | N.D. | N.D. | N.D. | N.D. | N.D. | N.D. | N.D. | N.D. | N.D. | N.D. | N.D. | N.D. | N.D. | N.D. | N.D. | N.D. | N.D. | N.D. | N.D. | N.D. | N.D. | 0/22 (0%) | — |
| PFBS | N.D. | N.D. | 0.510 | N.D. | N.D. | N.D. | N.D. | N.D. | N.D. | N.D. | N.D. | N.D. | N.D. | N.D. | N.D. | N.D. | N.D. | N.D. | N.D. | N.D. | N.D. | N.D. | 1/22 (5%) | 0.510 |
| PFHxS | 0.183 | N.D. | 0.402 | N.D. | N.D. | 0.167 | N.D. | N.D. | N.D. | N.D. | N.D. | N.D. | N.D. | N.D. | 0.121 | N.D. | N.D. | N.D. | 0.106 | N.D. | N.D. | N.D. | 5/22 (23%) | 0.167 |
| **PFOS** | **1.648** | **3.254** | **1.390** | **0.975** | **2.292** | **2.939** | **0.476** | **N.D.** | **0.172** | **N.D.** | **N.D.** | **0.935** | **0.347** | **3.323** | **1.292** | **2.002** | **N.D.** | **N.D.** | **0.270** | **N.D.** | **0.465** | **0.512** | **16/22 (73%)** | **1.133** |
| PFDS | N.D. | N.D. | N.D. | N.D. | N.D. | N.D. | N.D. | N.D. | N.D. | N.D. | N.D. | N.D. | N.D. | N.D. | N.D. | N.D. | N.D. | N.D. | N.D. | N.D. | N.D. | N.D. | 0/22 (0%) | — |
| PF-3,7-DMOA | N.D. | N.D. | N.D. | N.D. | N.D. | N.D. | N.D. | N.D. | N.D. | N.D. | N.D. | N.D. | N.D. | N.D. | N.D. | N.D. | N.D. | N.D. | N.D. | N.D. | N.D. | N.D. | 0/22 (0%) | — |
| PFPeS | N.D. | N.D. | N.D. | N.D. | N.D. | N.D. | N.D. | N.D. | N.D. | N.D. | N.D. | N.D. | N.D. | N.D. | N.D. | N.D. | N.D. | N.D. | N.D. | N.D. | N.D. | N.D. | 0/22 (0%) | — |
| PFHpS | N.D. | N.D. | N.D. | N.D. | N.D. | N.D. | N.D. | N.D. | N.D. | N.D. | N.D. | N.D. | N.D. | N.D. | N.D. | N.D. | N.D. | N.D. | N.D. | N.D. | N.D. | N.D. | 0/22 (0%) | — |
| PFNS | N.D. | N.D. | N.D. | N.D. | N.D. | N.D. | N.D. | N.D. | N.D. | N.D. | N.D. | N.D. | N.D. | N.D. | N.D. | N.D. | N.D. | N.D. | N.D. | N.D. | N.D. | N.D. | 0/22 (0%) | — |
| 4-2FTS | N.D. | N.D. | N.D. | N.D. | N.D. | N.D. | N.D. | N.D. | N.D. | N.D. | N.D. | N.D. | N.D. | N.D. | N.D. | N.D. | N.D. | N.D. | N.D. | N.D. | N.D. | N.D. | 0/22 (0%) | — |
| 6-2FTS | N.D. | N.D. | N.D. | N.D. | 0.333 | N.D. | N.D. | N.D. | N.D. | N.D. | 1.739 | N.D. | N.D. | N.D. | N.D. | N.D. | N.D. | N.D. | 1.841 | N.D. | N.D. | N.D. | 3/22 (14%) | 1.739 |
| 8-2FTS | N.D. | N.D. | N.D. | N.D. | N.D. | N.D. | N.D. | N.D. | N.D. | N.D. | N.D. | N.D. | N.D. | N.D. | N.D. | N.D. | N.D. | N.D. | N.D. | N.D. | N.D. | N.D. | 0/22 (0%) | — |
| NMeFOSAA | N.D. | N.D. | N.D. | N.D. | N.D. | N.D. | N.D. | N.D. | N.D. | N.D. | N.D. | N.D. | N.D. | N.D. | N.D. | N.D. | N.D. | N.D. | N.D. | N.D. | N.D. | N.D. | 0/22 (0%) | — |
| **6:2Cl-PFESA** | **1.978** | **3.301** | **0.827** | **0.456** | **2.169** | **2.374** | **0.243** | **N.D.** | **N.D.** | **N.D.** | **N.D.** | **N.D.** | **N.D.** | **N.D.** | **1.035** | **1.243** | **N.D.** | **N.D.** | **N.D.** | **N.D.** | **0.336** | **N.D.** | **10/22 (45%)** | **1.139** |
| 8-2Cl-PFESA | N.D. | N.D. | N.D. | N.D. | N.D. | N.D. | N.D. | N.D. | N.D. | N.D. | N.D. | N.D. | N.D. | N.D. | N.D. | N.D. | N.D. | N.D. | N.D. | N.D. | N.D. | N.D. | 0/22 (0%) | — |
| ADONA | N.D. | N.D. | N.D. | N.D. | N.D. | N.D. | N.D. | N.D. | N.D. | N.D. | N.D. | N.D. | N.D. | N.D. | N.D. | N.D. | N.D. | N.D. | N.D. | N.D. | N.D. | N.D. | 0/22 (0%) | — |
| HPFHpA | N.D. | N.D. | N.D. | N.D. | N.D. | N.D. | N.D. | N.D. | N.D. | N.D. | N.D. | N.D. | N.D. | N.D. | N.D. | N.D. | N.D. | N.D. | N.D. | N.D. | N.D. | N.D. | 0/22 (0%) | — |
| PFDoS | N.D. | N.D. | N.D. | N.D. | N.D. | N.D. | N.D. | N.D. | N.D. | N.D. | N.D. | N.D. | N.D. | N.D. | N.D. | N.D. | N.D. | N.D. | N.D. | N.D. | N.D. | N.D. | 0/22 (0%) | — |
| NEtFOSAA | N.D. | N.D. | N.D. | N.D. | N.D. | N.D. | N.D. | N.D. | N.D. | N.D. | N.D. | N.D. | N.D. | N.D. | N.D. | N.D. | N.D. | N.D. | N.D. | N.D. | N.D. | N.D. | 0/22 (0%) | — |
| HFPODA | N.D. | N.D. | N.D. | N.D. | N.D. | N.D. | N.D. | N.D. | N.D. | N.D. | N.D. | N.D. | N.D. | N.D. | N.D. | 0.621 | N.D. | N.D. | N.D. | N.D. | N.D. | N.D. | 1/22 (5%) | 0.621 |
| **HFPOTA** | **106.247** | **477.196** | **242.183** | **307.466** | **155.442** | **350.011** | **145.096** | **991.797** | **143.385** | **831.492** | **2071.364** | **1144.505** | **116.935** | **7012.889** | **1662.487** | **2082.976** | **1718.178** | **2555.556** | **192.986** | **1257.080** | **353.411** | **1495.517** | **22/22 (100%)** | **654.344** |

*Median of detected values only. Congeners shaded green (HFPOTA, PFOA, PFNA, PFUnDA, PFOS, 6:2Cl-PFESA) are the six core congeners (detection rate > 40%) used for the main statistical analyses. Sample IDs ending -N denote adjacent non-tumorous tissue and -T denote tumour tissue from the same patient.
