# Supplementary material for: Associations between legacy and emerging per and polyfluoroalkyl substances (PFAS) bioaccumulation and clinicopathological characteristics in breast cancer
Source: Front Public Health. 2026 Aug 18;14:1879297. doi: 10.3389/fpubh.2026.1879297 (PMC13529988; doi:10.3389/fpubh.2026.1879297)
Supplement: Supplementary file 3 [file Supplementary_file_3.docx]

**Table S2.** Analytical method and quality-control parameters for the six core PFAS congeners quantified in breast tissue.

**Method.** The 33-congener PFAS panel was quantified by a CMA-accredited laboratory (Shanghai Weipu Testing Technology Group) on a SCIEX ExionLC coupled to a SCIEX Triple Quad 5500+ operated in negative-ion electrospray with scheduled multiple-reaction monitoring (MRM). Quantification used external-standard calibration with a 12-point curve spanning 0.1–500 ng/mL; isotope-labelled internal standards were not applied. Quality control comprised a procedural blank (below detection for all analytes), a 10 ng/mL continuing calibration-verification (CCV) standard, and a 20 ng/mL matrix spike into a study sample. Across the six core congeners, CCV recovery was 106–113% and matrix-spike recovery was 85–100%, both within the conventional 70–130% acceptance window. Instrument detection limits were 2 ng/mL (HFPOTA), 0.2 ng/mL (PFODA) and 0.1 ng/mL (all remaining analytes).

| **Congener** | **MRM transition (Q1 > Q3, m/z)** | **Retention time (min)** | **Instrument LOD (ng/mL)** | **CCV recovery (10 ng/mL, %)** | **Matrix-spike recovery (%)** |
| --- | --- | --- | --- | --- | --- |
| HFPOTA | 184.9 > 119.0 | 8.8 | 2 | 112 | 85 |
| PFOA | 413.0 > 369.0 | 7.3 | 0.1 | 108 | 95 |
| PFNA | 463.0 > 419.0 | 8.3 | 0.1 | 106 | 95 |
| PFUnDA | 563.0 > 519.0 | 10.1 | 0.1 | 113 | 100 |
| PFOS | 499.0 > 80.0 | 9.5 | 0.1 | 109 | 92 |
| 6:2Cl-PFESA | 531.98 > 352.0 | 10.1 | 0.1 | 108 | 85 |

*CCV, continuing calibration verification; LOD, limit of detection; MRM, multiple-reaction monitoring.*

*Quantifier MRM transitions and per-standard calibration data are taken from the instrument raw-data report; matrix-spike recovery = (spiked − unspiked concentration) / 20 ng/mL added, measured in study sample 727624T.*

*Method limits of detection/quantitation in ng/g tissue depend on the wet tissue mass extracted; instrument LODs above are given in ng/mL of final extract.*

*The full 33-congener MRM list and calibration records are available from the laboratory on request.*
