# Supplementary material for: Associations between legacy and emerging per and polyfluoroalkyl substances (PFAS) bioaccumulation and clinicopathological characteristics in breast cancer
Source: Front Public Health. 2026 Aug 18;14:1879297. doi: 10.3389/fpubh.2026.1879297 (PMC13529988; doi:10.3389/fpubh.2026.1879297)
Supplement: Supplementary file 4 [file Supplementary_file_4.docx]

**Table S3.** Paired tumour-versus-adjacent-non-tumorous effect sizes for the six core PFAS congeners (two-sided exact Wilcoxon signed-rank test).

| **Congener** | **n (pairs)** | **Fold-change (tumour/ANT)** | **95% CI** | **Raw p** | **BH q** |
| --- | --- | --- | --- | --- | --- |
| HFPOTA | 11 | 3.02 | 1.49–6.84 | 0.007 | 0.041 |
| PFNA | 11 | 1.88 | 1.15–4.28 | 0.016 | 0.047 |
| PFUnDA | 11 | 1.75 | 1.03–4.13 | 0.023 | 0.047 |
| 6:2Cl-PFESA | 4 | 1.41 | not estimable | 0.125 | 0.188 |
| PFOA | 11 | 1.36 | 0.77–2.93 | 0.413 | 0.496 |
| PFOS | 11 | 1.15 | 0.48–3.50 | 0.732 | 0.732 |

*ANT, adjacent non-tumorous tissue; CI, confidence interval; BH, Benjamini–Hochberg false-discovery-rate correction across the six core congeners.*

*Fold-change was obtained by back-transforming the Hodges–Lehmann pseudomedian (location-shift estimate) of the paired log2 differences (2^HL); the 95% CI is the exact Wilcoxon confidence interval. Non-detects were substituted with LOD/√2 and retained, so that all 11 patients (or the 4 with measurable paired 6:2Cl-PFESA values) contribute to each paired test.*

*For 6:2Cl-PFESA only four patients had measurable paired values, so the confidence interval is not estimable and the result is reported descriptively.*
